# lncSNHG3 drives breast cancer progression by epigenetically increasing CSNK2A1 expression level

**DOI:** 10.18632/aging.204824

**Published:** 2023-06-21

**Authors:** Zhenlin Nie, Mu Xu, Linpeng Zhou, Bei Pan, Tao Xu, Bangshun He, Shukui Wang

**Affiliations:** 1 Department of Laboratory Medicine, Nanjing First Hospital, Nanjing Medical University, Nanjing 210006, Jiangsu, China; 2 Division of Clinical Pharmacy, Nanjing First Hospital, China Pharmaceutical University, Nanjing 210006, Jiangsu, China; 3 Jiangsu Cancer Personalized Medicine Collaborative Innovation Center, Nanjing 210029, Jiangsu, China; 4 General Clinical Research Center, Nanjing First Hospital, Nanjing Medical University, Nanjing 210006, Jiangsu, China

**Keywords:** SNHG3, CSNK2A1, miR-485-5p, HuR, breast cancer

## Abstract

Mounting evidence demonstrates that long noncoding RNAs (lncRNAs) have critical roles in the initiation and progression of cancer. Here, we report that small nucleolar RNA host gene 3 (SNHG3) is a key regulator of breast cancer progression. We analyzed RNA sequencing data to explore abnormally expressed lncRNAs in breast cancer. The effects of SNHG3 on breast cancer were investigated via *in vitro* and *in vivo* assays (CCK-8 assay, colony formation assay, flow cytometry assay, EdU assay, xenograft model, immunohistochemistry, and Western blot). The mechanism of SNHG3 action was explored through bioinformatics, RNA fluorescence *in situ* hybridization, luciferase reporter assay, RNA pull-down assay, chromatin immunoprecipitation assay and RNA immunoprecipitation assay. We found that SNHG3 expression was upregulated in breast cancer tissues and that its high expression level was associated with poor survival. We also found that high SNHG3 expression was partly induced by STAT3. Moreover, SNHG3 knockdown significantly repressed breast cancer cell growth both *in vitro* and *in vivo*. In the cytoplasm, SNHG3 facilitated the expression of Casein kinase II-A1 (CSNK2A1) by absorbing miR-485-5p and recruiting the HuR protein, participating in the malignant progression of breast cancer. Taken together, our study reveals a SNHG3-based regulatory network, which plays an oncogenic role in breast cancer and suggests that SNHG3 may serve as a potential target for the diagnosis and treatment of breast cancer.

## INTRODUCTION

Breast cancer (BC) is a prevalent malignancy affecting women worldwide. It is a diverse condition encompassing various subtypes with unique characteristics regarding histopathology, molecular changes, and clinical progression [1, 2]. Despite significant progress in diagnosis and treatment, the prognosis for BC patients remains unfavorable. This can be attributed to the frequent occurrence of metastasis and recurrence [3]. Consequently, it is crucial to uncover novel biomarkers that can aid in predicting prognosis and gain a better consideration of the precise mechanisms at the molecular level driving BC malignancy. These discoveries hold the potential to offer enhanced treatment choices for individuals affected by BC.

Long non-coding RNAs (lncRNAs) are a category of RNA molecules that surpass 200 nucleotides in length and do not possess the capability to encode proteins. Their importance in regulating fundamental tumor-associated mechanisms such as epithelial-mesenchymal transition (EMT), the progression of invasion and metastasis, proliferation, and drug resistance has been well established [4–7]. Multiple investigations have recently provided evidence indicating that aberrantly expressed long non-coding RNAs (lncRNAs) act as tumor suppressors or oncogenes and played significant roles in BC progression. A clear illustration of this occurrence can be observed with the long non-coding RNA BCRT1, which can competitively bind with miR-1303, thereby obstructing its target gene PTBP3 degradation. As a result, BCRT1 functions as an oncogenic factor in BC, promoting tumor growth and progression. Furthermore, the overexpression of lncRNA BCRT1 can induce the M2 polarization of macrophages through exosome-mediated mechanisms, ultimately contributing to the advancement of BC [8]. The polypeptide ASRPS (a small regulatory peptide of STAT3), encoded by a long non-coding RNA (lncRNA), demonstrates inhibitory effects on angiogenesis in triple-negative BC [9]. By analyzing transcriptome data from The Cancer Genome Atlas database, we identified a marked upregulation in the expression of small nucleolar RNA host gene 3 (SNHG3) within BC tissues. However, the precise function and underlying process of SNHG3 in BC remains predominantly unknown.

SNHG3, located at 1q35.3, has been implicated in advancing multiple tumor types, including `bladder cancer, hepatocellular carcinoma, and colorectal cancer. Consequently, its association with these malignancies contributes to the unfavorable prognosis of patients afflicted by these tumors [10–12]. However, the precise biological roles and mechanisms through which SNHG3 contributes to breast cancer are poorly comprehended. CSNK2A1, a catalytic subunit of Casein kinase II (CSNK2), has been recognized as participating in different cancer cells’ invasive and migratory processes by triggering epithelial-mesenchymal transformation (EMT) [13–15]. Consequently, investigating the function and regulatory mechanisms of CSNK2A1 holds immense significance in clinical cancer treatment.

Here, we report the biological functions of SNHG3 as a lncRNA abnormally overexpressed in BC that is implicated in the regulation of malignant progression and poor prognosis. In addition, we proved that the transcription factor STAT3 activates SNHG3 transcription in BC. Moreover, our findings indicate that SNHG3 exhibits promising potential as a novel biomarker for diagnosing and treating BC.

## RESULTS

### SNHG3 expression is upregulated in BC, and higher SNHG3 expression levels predict a worse prognosis of BC patients

**Figure 1:**
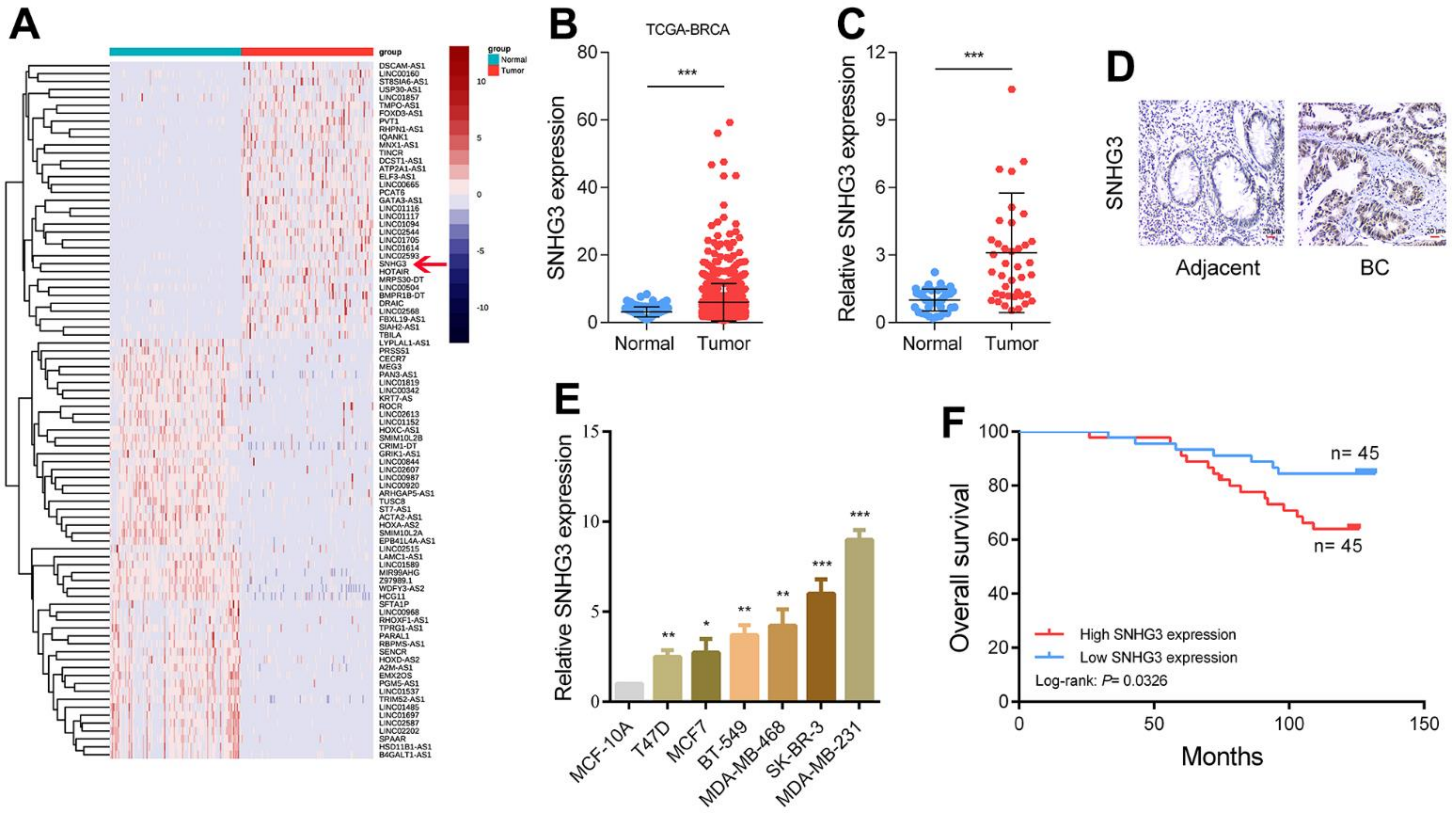
**SNHG3 expression is upregulated in BC and high SNHG3 expression predicts poor prognosis.** (**A**) Hierarchical cluster heat map of aberrantly expressed lncRNAs in BC generated from RNA sequencing data from the TCGA database. Red in the heat map denotes upregulation, while blue denotes downregulation. The red line indicates SNHG3. (**B**) Expression of SNHG3 in TCGA BC cohorts. (**C**) qRT-PCR analysis of SNHG3 expression in 40 pairs of BC and corresponding normal tissues. (**D**) ISH analysis of SNHG3 expression in BC and corresponding normal tissues. (**E**) SNHG3 expression in BC cell lines (T470, MCF7, BT-549, MDA-MB-468, SK-BR-3 and MDA-MB-231) compared with normal breast epithelial cell line MCF-10A detected by qRT-PCR. (**F**) Kaplan–Meier survival analysis of BC patients’ overall survival based on their SNHG3 expression.

Initially, we analyzed the transcriptome data obtained from the TCGA database to identify dysregulated long non-coding RNAs (lncRNAs) in BC (Figure 1A). Among the dysregulated lncRNAs, SNHG3 was significantly elevated, and its abundance was relatively high (Figure 1B). The levels of SNHG3 expression were evaluated in BC tissues compared with adjacent normal tissues through qPCR and ISH experiments. The results from these experiments confirmed the findings derived from the sequencing data (Figure 1C, 1D and Supplementary Figure 1A). In addition, BC cell lines demonstrated a significant overexpression of SNHG3 in comparison to MCF-10A normal human breast epithelial cells (Figure 1E). We also analyzed the expression levels of SNHG3 in BC tissues and the clinical characteristics of BC patients and found that higher SNHG3 expression levels indicated a worse prognosis of patients. However, no significant variation was detected in the expression level of SNHG3 among BC tissues with distinct tumor stages and lymph node metastasis status (Figure 1F and Supplementary Figure 1B, 1C).

### STAT3 activates SNHG3 transcription in BC

**Figure 2:**
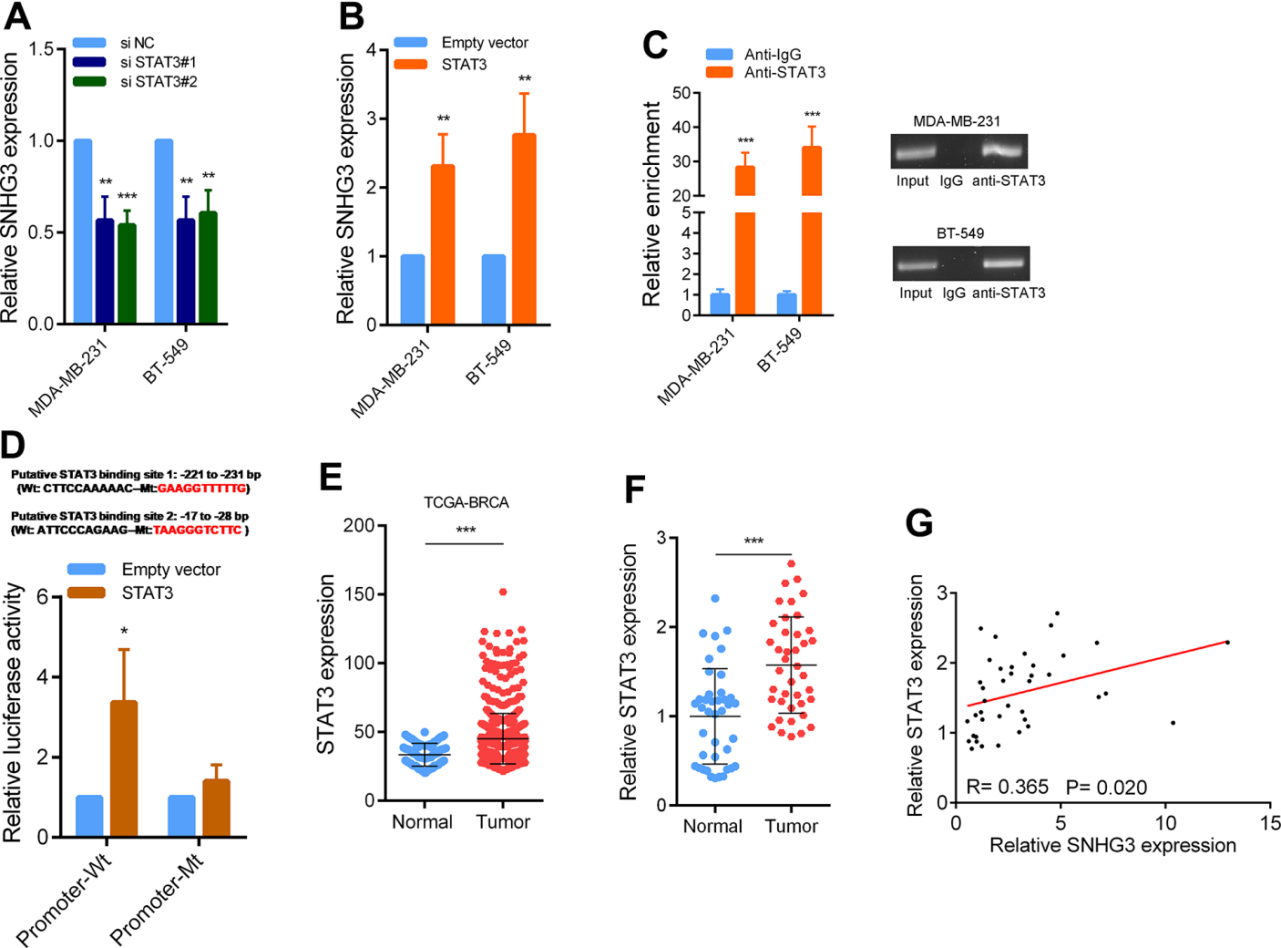
**STAT3 activation induce high SNHG3 expression in BC.** (**A**, **B**) SNHG3 expression was detected by qRT-PCR in MDA-MB-231 and BT549 cells transfected with the STAT3 siRNAs or STAT3 overexpression vector. (**C**) ChIP assays were performed to detect STAT3 occupancy in the SNHG3 promoter region. (**D**) Luciferase reporter assays were used to determine the STAT3 binding sites on the SNHG3 promoter region. (**E**) Expression of STAT3 in TCGA BC cohorts. (**F**) qRT-PCR analysis of STAT3 expression in 40 pairs of BC and corresponding normal tissues. (**G**) The correlation between the STAT3 and SNHG3 expression levels were analyzed in 40 paired BC samples.

To determine the specific transcription factor responsible for the upregulation of SNHG3 expression in BC, we employed JASPAR tools for predicting potential transcription factors capable of binding to the SNHG3 promoter region. Among them, STAT3 attained the highest score, indicating its possible involvement in regulating SNHG3 expression. We then determined whether STAT3 can affect SNHG3 expression in BC. The findings demonstrated a significant reduction in SNHG3 expression levels following STAT3 knockdown in BC cells, while the overexpression of STAT3 resulted in a notable increase in SNHG3 expression (Figure 2A, 2B and Supplementary Figure 2A). Furthermore, the findings from ChIP experiments provided evidence that STAT3 directly interacts with the promoter region of SNHG3. Additionally, luciferase reporter assays confirmed the binding sequence (Figure 2C, 2D). This finding was supported by the sequencing data obtained from BC tissues in the TCGA database, which indicated significant upregulation of STAT3 expression in BC (Figure 2E). To confirm the data, we also measured STAT3 expression in 40 pairs of BC and adjacent tissues by qPCR, and the results were consistent with the sequencing results (Figure 2F). Additionally, correlation analysis showed that SNHG3 and STAT3 expression levels were significantly positively correlated in BC tissues (Figure 2G).

### SNHG3 affects the proliferation and metastatic ability of BC cells *in vitro*

**Figure 3:**
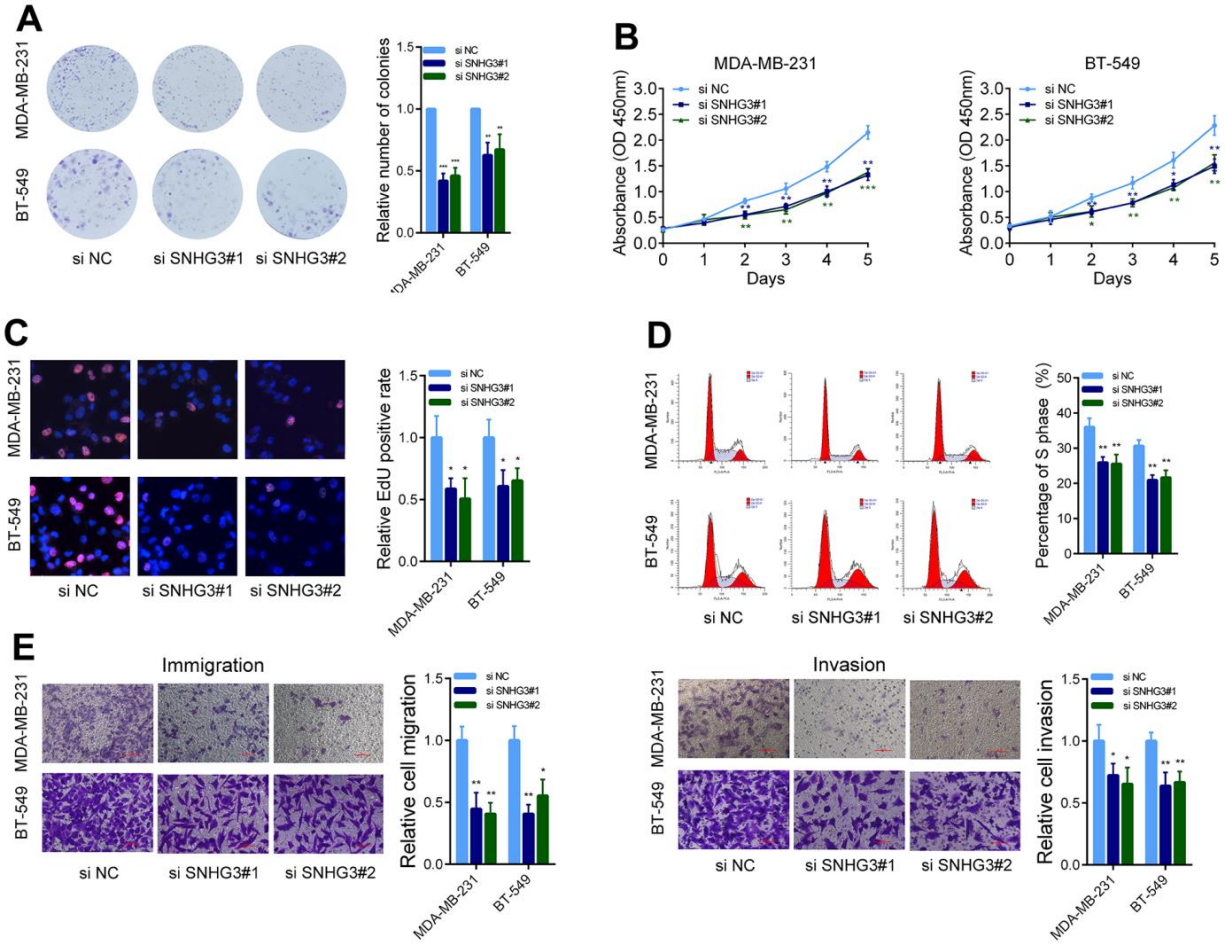
**SNHG3 affects BC cell growth and metastasis *in vitro*.** (**A**) MDA-MB-231 and BT549 cells transfected with SNHG3 siRNAs were seeded into 6-well plates. The number of colonies was counted on the 14th day after seeding. (**B**) MDA-MB-231 and BT549 cells transfected with the SNHG3 siRNAs were subjected to the CCK-8 assay. (**C**) EdU assays were used to determine the cell proliferation ability of SNHG3 siRNAs transfected cells. (**D**) Flow cytometric cell cycle distribution assays to detect the proportion of BC cells in the G1, S, and G2/M phases after the transfection of SNHG3 siRNAs. (**E**) Transwell assays were used to determine the invasion and migration abilities of SNHG3 siRNAs transfected cells.

**Figure 4:**
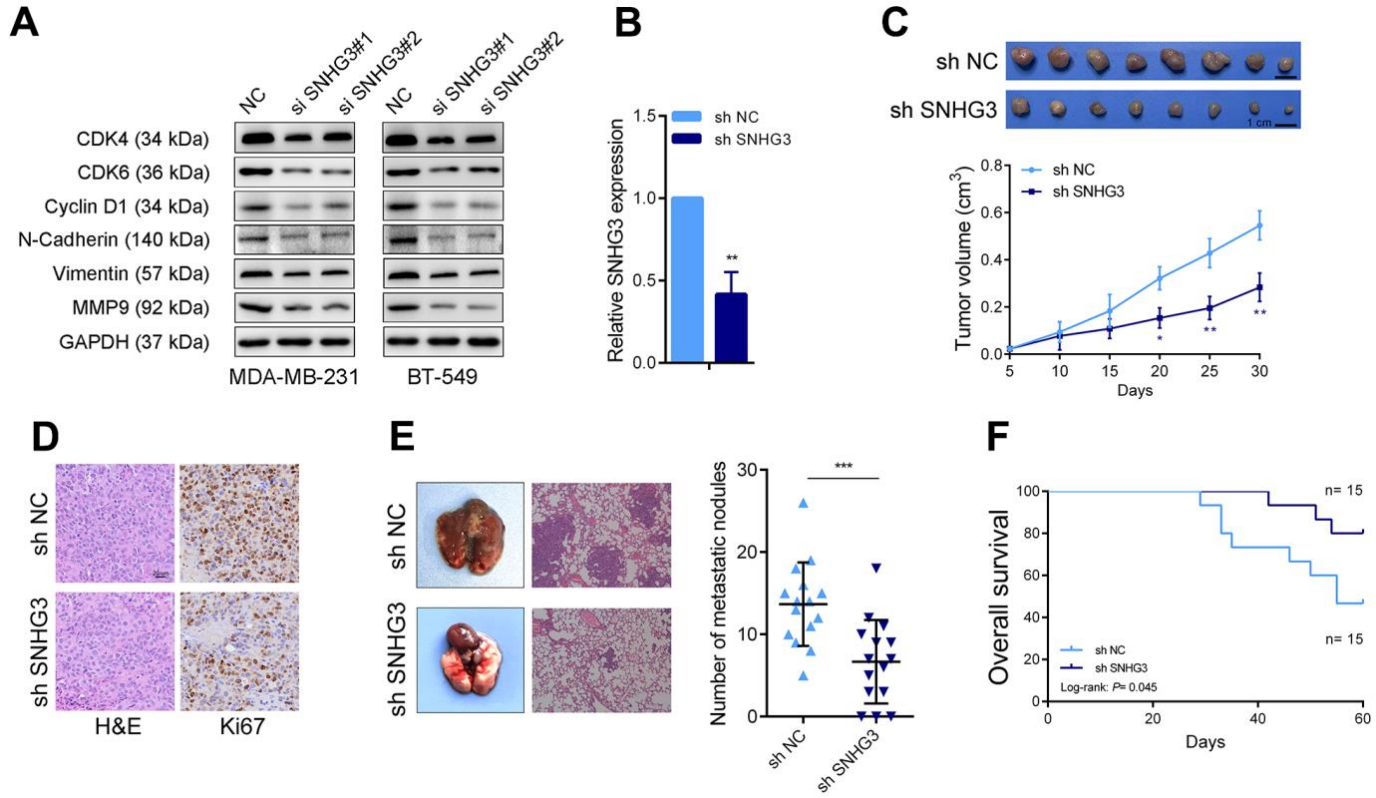
**SNHG3 promotes BC growth and metastasis *in vivo*.** (**A**) Cell cycle-related proteins CyclinD1, CDK4, and CDK6 and the metastasis-related proteins N-cadherin, vimentin, and MMP9 were detected by western blotting after SNHG3 knockdown. (**B**) SNHG3 expression was detected by qRT-PCR in SNHG3 stable knocked down MAD-MB-231 cells. (**C**) Representative image of tumors formed in nude mice and tumor volume growth curves of different groups. (**D**) Representative images for HE-staining, Ki67 immunostaining of tumor samples from the different groups. (**E**) Left panel, representative images of the gross lesion in lung tissues and hematoxylin and eosin (HE) staining of metastatic nodules in the lungs from the different groups. Right panel, the statistical result of metastatic nodule numbers in the lungs from the different groups. (**F**) Survival analysis of the nude mouse from different groups.

We then evaluated the effects of SNHG3 expression on BC cell biological behavior. The colony formation assays demonstrated that the suppression of SNHG3 significantly diminished the ability of BC cells to form colonies (Figure 3A). Following the silencing of SNHG3 expression, the CCK-8 assay revealed a substantial decline in the expansion of BC cells (Figure 3B). Additionally, the EdU assay, in combination with flow cytometry cell cycle analysis, demonstrated a noteworthy decrease in the proportion of proliferating BC cells upon SNHG3 knockdown (Figure 3C, 3D). Additionally, Transwell experiments revealed that SNHG3 inhibition significantly decreased BC cells’ capacity for migration and invasion (Figure 3E). Additionally, the Western blot analysis revealed that the knockdown of SNHG3 significantly reduced the protein levels of proteins involved in the cell cycle (CDK6, CDK4, and Cyclin D1) as well as cancer metastasis (N-Cadherin, Vimentin, and MMP9) (Figure 4A).

### SNHG3 promotes BC cell proliferation and metastasis *in vivo*

For the determination of SNHG3 effects on BC cells *in vivo*, we constructed an SNHG3 stably knockdown MDA-MB-231 cell line (Figure 4B). In the nude mouse xenograft tumor model; it was observed that the suppression of SNHG3 significantly impeded the enlargement of BC cells. The volume of xenograft tumors formed by SNHG3 knockdown cells was notably smaller compared to the control group (Figure 4C). Additional information was obtained from the ISH assays, which showed that the SNHG3 knockdown group had a substantially lower positive rate of Ki67 than the control group (Figure 4D). Furthermore, in the lung metastasis model where tumor cells were injected into the tail vein, knocking down SNHG3 resulted in a substantial decline in the metastatic capability of BC cells. The SNHG3 knockdown group exhibited a significantly lower number of metastatic liver nodules than the control group, accompanied by a notable extension in the survival time of mice within the SNHG3 knockdown group (Figure 4E, 4F).

### SNHG3 regulates CSNK2A1 expression in BC

**Figure 5:**
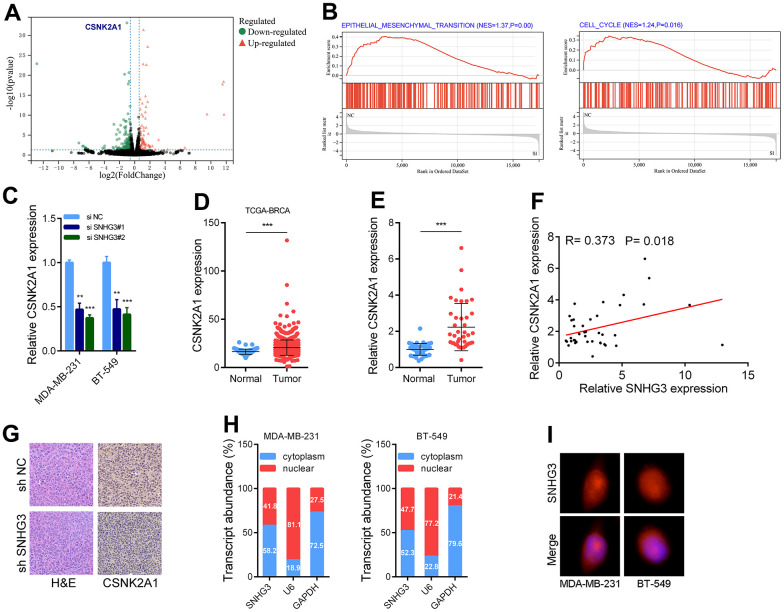
**SNHG3 regulates CSNK2A1 expression in BC.** (**A**) Volcano plot showing aberrantly expressed genes after knockdown of SNHG3 in MDA-MB-231. (**B**) GSEA results were plotted to visualize the pathways related to SNHG3 regulated genes. (**C**) CSNK2A1 expression was detected by qRT-PCR in MDA-MB-231 and BT549 cells transfected with the SNHG3 siRNAs. (**D**) Expression of CSNK2A1 in TCGA BC cohorts. (**E**) qRT-PCR analysis of CSNK2A1 expression in 40 pairs of BC and corresponding normal tissues. (**F**) The correlation between the CSNK2A1 and SNHG3 expression levels were analyzed in 40 paired BC samples. (**G**) Representative images for HE-staining, CSNK2A1 immunostaining of tumor samples from the different groups. (**H**) Relative SNHG3 expression levels in nuclear and cytosolic fractions of MDAMB-231 and BT549 cells. Nuclear controls: U6, cytosolic controls: GAPDH. (**I**) Representative FISH images showed the expression of SNHG3 in MDA-MB-231 and BT549 cells (red). Nuclei were stained by DAPI (blue).

After knocking down SNHG3 in BC cells, we used transcriptome sequencing to identify target genes of SNHG3. The GSEA [16] results showed that the EMT pathway and cell cycle-related pathways were significantly altered in BC cells following SNHG3 knockdown (Figure 5B). One of the target genes was the oncogene CSNK2A1, whose expression level was significantly downregulated, and this result was verified by PCR experiments (Figure 5A, 5C). Furthermore, a notable rise in the expression of CSNK2A1 in BC tissues was observed, with its expression level displaying a positive correlation with SNHG3 expression (Figure 5D–5F). ISH assays conducted on transplanted tumors also demonstrated a significant reduction in the expression of CSNK2A1 following SNHG3 knockdown. Hence, we identified CSNK2A1 as a target gene of SNHG3 for further investigation (Figure 5G). Additionally, we found that SNHG3 was distributed in both the cytoplasm and nucleus of BC cells (Figure 5H, 5I).

### SNHG3 promotes CSNK2A1 expression by absorbing miR-485-5p and recruiting the HuR protein

**Figure 6:**
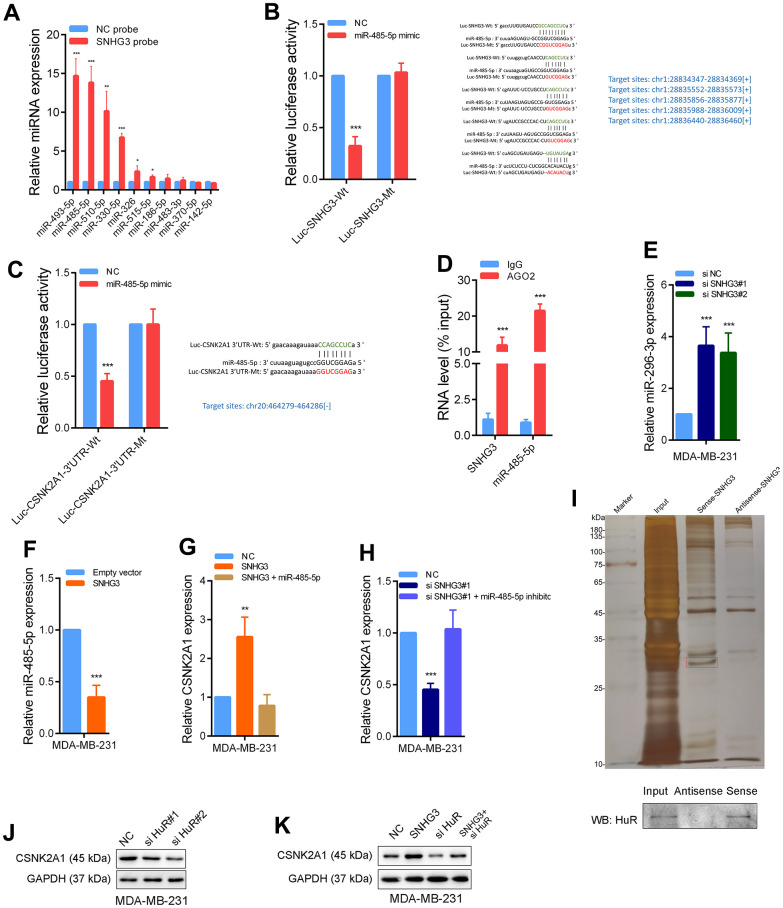
**SNHG3 promotes CSNK2A1 expression through interaction of miR-485-5p and HuR protein.** (**A**) The relative expression of candidate microRNAs which could potentially bind to SNHG3 were quantified by qRT-PCR after the biotinylated-SNHG3 pulldown assays in MDA-MB-231 cells. (**B**) Dual luciferase reporter assays were conducted with wild-type and mutant-type (putative binding sites for miR-485-5p were mutated) luciferase report vectors of SNHG3 and miR-485-5p. Right panel, sequence alignment of miR-485-5p and its predicted binding sites (green) of SNHG3. Predicted microRNA target sequence (blue) in SNHG3 (Luc-SNHG3-wt) and positions of mutated nucleotides (red) in SNHG3 (Luc-SNHG3-mt). (**C**) Dual luciferase reporter assays were conducted with wild-type and mutant-type (putative binding sites for miR-485-5p were mutated) luciferase report vectors of CSNK2A1 3’UTR and miR-485-5p. (**D**) RNA immunoprecipitation with an anti-Ago2 antibody was used to assess endogenous Ago2 binding to RNA, IgG was used as the control. The levels of SNHG3 and miR-485-5p were determined by qRT–PCR and presented as fold enrichment in Ago2 relative to input. (**E**, **F**) MiR-485-5p expression was detected by qRT-PCR after SNHG3 was silenced or overexpressed. (**G**, **H**) CSNK2A1 expression was detected by qRT-PCR in MDA-MB-231 cells with indicated treatment. (**I**) Representative image of silver-stained PAGE gels showing separated proteins that were pulled down using biotin-labeled SNHG3. A red frame indicates HuR. (**J**, **K**) CSNK2A1 expression was detected by western blot in MDA-MB-231 cells with indicated treatment.

For the investigation of the underlying molecular mechanism by which SNHG3 regulates CSNK2A1, since most SNHG3 is in the cytoplasm, we synthesized biotinylated SNHG3 probes. We performed RNA pull-down experiments to identify microRNAs directly bound to SNHG3. The finding indicates that miR-485-5p had the highest binding abundance with SNHG3. Bioinformatics tools predicted that it could bind not only to SNHG3 but also to the 3’UTR of CSNK2A1 (Figure 6A). For additional confirmation of the SNHG3-miR-485-5p interaction, we developed a luciferase reporter plasmid (Luc-SNHG3-Wt) with the expected binding sites, as well as a corresponding mutant plasmid (Luc-SNHG3-Mt) with the binding site mutated. The luciferase reporter assays provided compelling evidence that the upregulation of miR-485-5p resulted in a substantial decline in luciferase intensity in cells transfected with the wild-type plasmid Luc-SNHG3-Wt. However, transfection of the mutant-type plasmid Luc-SNHG3-Mt did not impact the luciferase intensity in cells. (Figure 6B). Additionally, we designed a luciferase reporter plasmid to monitor miR-485-5p’s interaction with the 3’UTR of CSNK2A1. Similarly, the overexpression of miR-485-5p markedly reduced the luciferase intensity of the wild-type plasmid Luc-CSNK2A1-3’UTR-Wt. At the same time, it had no impact on the luciferase intensity of the mutant plasmid Luc-CSNK2A1-3’UTR-Mt (Figure 6C). Additionally, the RNA immunoprecipitation (RIP) assays demonstrated direct binding of the AGO2 protein to both miR-485-5p and SNHG3 (Figure 6D). In addition, knocking down SNHG3 significantly increased the expression level of miR-485-5p and reduced the expression level of CSNK2A1, while overexpression of SNHG3 significantly reduced the expression level of miR-485-5p and increased the expression level of CSNK2A1. Furthermore, overexpression of SNHG3 while overexpressing miR-485-5p weakened the increase in CSNK2A1, and knocking down SNHG3 while inhibiting miR-485-5p weakened the decrease in CSNK2A1 expression level (Figure 6E–6H and Supplementary Figure 3A). Besides, correlation analysis showed that SNHG3 and miR-485-5p expression levels were significantly negatively correlated in BC tissues (Supplementary Figure 3B).

Moreover, the findings from the RNA pull-down assays provided evidence that SNHG3 directly binds to the HuR protein (Figure 6I). It is reasoned that SNHG3 may enhance the stability of CSNK2A1 mRNA by recruiting HuR. The results demonstrated a notable reduction in the CSNK2A1 expression level following the knockdown of HuR. The knockdown of HuR notably attenuated the increase in CSNK2A1 expression induced by SNHG3 overexpression (Figure 6J, 6K). In conclusion, these data strongly suggest that SNHG3 advances the expression of CSNK2A1 in BC by interacting with both miR-485-5p and the HuR protein.

### SNHG3 promotes BC progression depending on its regulation of CSNK2A1

**Figure 7:**
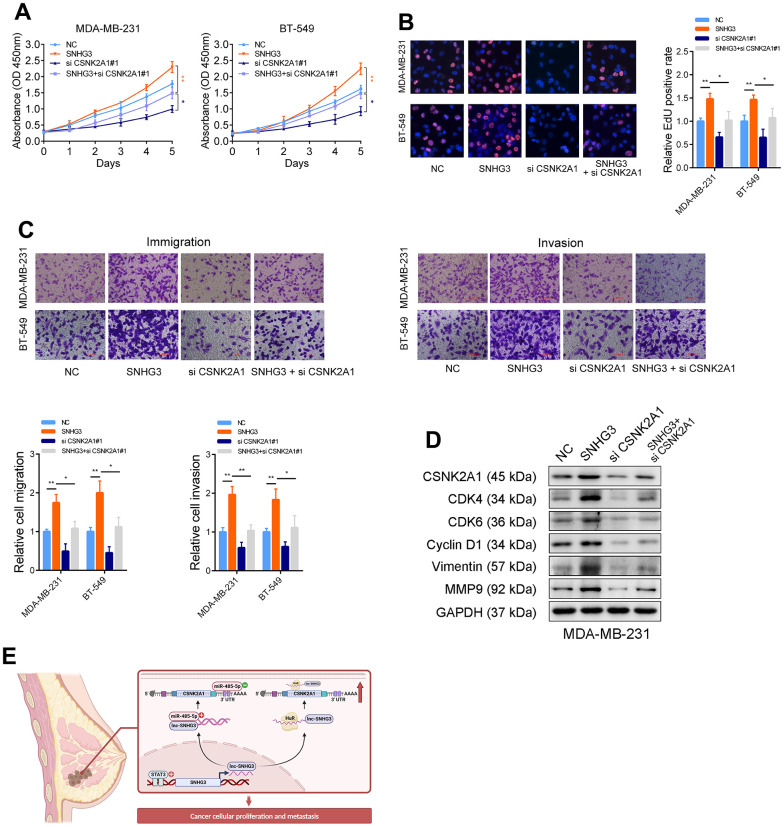
**Tumor-promoting functions of SNHG3 is dependent on CSNK2A1.** (**A**) CCK-8 assays demonstrated that overexpression of SNHG3 promoted cancer cell growth. CSNK2A1 knockdown could abolish growth promotion caused by SNHG3. (**B**) EdU assays showed that CSNK2A1 knockdown abolished the increased proliferation rates of MDA-MB-231 cells caused by SNHG3. (**C**) Transwell assays demonstrated that CSNK2A1 knockdown abolished the increased abilities of migration and invasion caused by SNHG3. (**D**) The CSNK2A1, cell cycle-related proteins, and metastasis-related proteins were detected by western blotting with indicated treatment. (**E**) Schematic of the proposed mechanism of SNHG3 in breast cancer cells.

Rescue experiments were carried out to determine how SNHG3’s regulation of CSNK2A1 contributes to the progression of BC. The findings from CCK-8, EdU, and Transwell experiments demonstrated that the overexpression of SNHG3 augmented BC cells’ growth and migration capabilities. However, when CSNK2A1 was simultaneously knocked down alongside SNHG3 overexpression, the effects of SNHG3 overexpression were significantly mitigated (Figure 7A–7C). Moreover, the findings of Western blot indicated that the knockdown of CSNK2A1 attenuated the alterations in migration- and growth-related proteins resulting from SNHG3 overexpression (Figure 7D). These findings imply that SNHG3 promotes the progression of BC depending on its upregulation of CSNK2A1 (Figure 7E).

## DISCUSSION

BC is a significant public health concern threatening women’s health, and there is still a lack of effective therapeutic targets [17, 18]. lncRNAs are crucial in regulating various malignancies, including BC, and have been extensively investigated as potential targets for early diagnosis and treatment interventions [19, 20]. Therefore, there is an urgent need to explore the mechanism of lncRNAs in BC. SNHG3, a newly identified long non-coding RNA, has emerged as a significant player in the pathogenesis and advancement of various cancer types. Extensive research has revealed that SNHG3 expression is commonly elevated in cancerous tissues, correlating with aggressive tumor characteristics and unfavorable patient prognoses [21, 22]. However, the specific functions and underlying mechanisms of SNHG3 in BC progression remain inadequately elucidated. The present study discovered a significant upregulation of lncRNA SNHG3 in BC tissues and cells. Moreover, we observed that heightened SNHG3 expression amplified BC cells’ proliferation and metastatic capabilities both *in vivo* and *in vitro*. In our investigation, we elucidated the mechanism by which STAT3 activated the transcription of SNHG3. Additionally, SNHG3 acted as a mediator for the expression of CSNK2A1 by sequestering miR-485-5p and recruiting HuR protein, thereby participating in the malignant progression of BC.

lncRNAs can regulate target gene expression through a variety of mechanisms [23]. Our findings demonstrate that SNHG3 is predominantly localized in the cytoplasm.

Previous research has suggested that cytoplasmic long non-coding RNAs (lncRNAs) can function as molecular decoys, sequestering microRNAs (miRNAs) and thereby modulating the expression of miRNA target genes [24]. This hypothesis, proposed initially by Pier Paolo Pandolfi, proposes that RNA transcripts, including mRNAs and lncRNAs, competitively interact with shared microRNA response elements (MREs). This competition forms a complex crosstalk network, enabling the regulation of gene expression at the posttranscriptional level [25]. The complex interplay mentioned above also occurs within the cancer advancement framework, especially in the processes of invasion and metastasis. For example, in the case of BC, there is a positive association between the lncRNA HOTAIR and metastasis [26]; HOTAIR acts as a miR-20a-5p sponge, significantly impacting the migration and invasion of tumor cells by modulating the HOTAIR/miR-20a-5p/HMGA2 pathway [27].

Similarly, the upregulation of LINC00461 enhances BC cell invasion by acting as a sponge for miR-30a-5p to regulate integrin [28]. In addition, the lncRNA BCRT1 enhances the invasion of BC by working as a ceRNA for miR-1303. As a result, it counteracts the inhibitory impact of miR-1303 on its target gene, PTBP38 [8]. Our investigation revealed that SNHG3 possesses multiple binding sites for miR-485-5p, and alterations in SNHG3 expression can impact the abundance of miR-485-5p. MiR-485-5p has been identified as a tumor suppressor gene in various cancers. For instance, Kang et al. demonstrated that miR-485-5p targets flotillin-1, thus inhibiting gastric cancer progression [29]. Similarly, Chen et al. proved that miR-485-5 target HMGA2, thus suppresses bladder cancer metastasis [30]. Moreover, miR-485-5p is significantly downregulated in BC, exerting its tumor-suppressive effects by regulating PGC-1α and promoting survival [31, 32].

Furthermore, this research also indicates that miR-485-5p targets CSNK2A1 in BC, and SNHG3 can influence the expression of CSNK2A1 through its interaction with miR-485-5p. Notably, CSNK2A1 has been found as an oncogene in multiple tumor types. Consistently, upregulation of CSNK2A1 expression has been observed in various cancer types, including the colorectal cancer [33], stomach cancer [34], head and neck cancer [35], kidney cancer [36, 37], and prostate cancer [38], compared to noncancerous tissues. Furthermore, the prognostic relevance of CSNK2A1 has been reported in multiple cancer types, including colorectal cancer [33], gastric cancer [34], clear cell renal cell carcinoma [37] and prostatic cancer [38]. In cases of gastric carcinomas, the immunohistochemical detection of CSNK2A1 expression has been found as a self-regulating prognostic factor associated with recurrence-free survival (RFS) and reduced overall survival in cancer patients [34]. In cases of gastric carcinomas, the immunohistochemical detection of CSNK2A1 expression has been found as a self-regulating prognostic factor associated with recurrence-free survival (RFS) and reduced overall survival in cancer patients [37]. These conclusions strongly reveal that CSNK2A1 has a substantial role in advancing human cancers and holds the potential as a prognostic marker for various types of malignancies. Multiple studies have reported the upregulation of CSNK2A1, which promotes BC progression. The decreased expression of CSNK2A1 in BC extensively reduced cell viability and clonal survival, reduced mRNA and protein expression of relevant factors, and induced alterations associated with cell death [39]. The phosphorylation of SIRT6 mediated by CSNK2A1 has been recognized as a regulatory mechanism governing proliferation and invasion in BC. Depletion of CSNK2A1 in BC cells led to decreased proliferation and invasion, while its overexpression was linked to increased proliferation and invasion [40]. These studies collectively highlight the crucial role of CSNK2A1 in promoting BC cell survival, aligning with our findings.

In addition to acting as a molecular sponge, SNHG3 was identified to bind to the HuR protein directly, which is an RNA-stabilizing protein, and its role in tumors has been widely reported [41, 42]. Several studies have found that lncRNAs or circRNAs can stabilize their targets by recruiting the HuR protein. Zhou et al. found that VPS9D1-AS1 was able to bind to the HuR protein and thereby influence the stability and expression of CDK4 mRNA, thus impacting HCC cell proliferation [43]. In CRC development, circRHOBTB3 binds to HuR and exerts suppressive effects. Normally, HuR binds to the 3’UTR of target mRNAs to facilitate their stabilization, whereas the interaction between circRHOBTB3 and HuR degrades HuR to reduce the expression level of the downstream target PTBP1 [44]. Here, we found that SNHG3 can recruit HuR to maintain the stability of CSNK2A1 and promote its expression. Our study reveals a novel regulatory pathway of CSNK2A1 in BC. Furthermore, rescue experiments confirmed that the protumor role of SNHG3 is dependent on its regulation of CSNK2A1.

## CONCLUSIONS

Our study shows that SNHG3 is elevated in BC and promotes BC progression by enhancing CSNK2A1 expression. We reveal a novel molecular regulatory pathway in BC and indicate that SNHG3 is a potential therapeutic target for BC.

## MATERIALS AND METHODS

### Tissue samples

Nanjing First Hospital provided forty matched pairs of BC tissues and their corresponding adjacent tissues stored in liquid nitrogen. The above patients received radical BC surgery and were not treated with radiotherapy before surgery. Survival analysis was done using a breast tumor tissue microarray from the Shanghai Outdo Biotech Co. (Shanghai, China). Before inclusion in the study, every patient in the cohort provided informed consent, and the research protocol received approval from the Ethics Committee on Human Research at Nanjing First Hospital, Nanjing Medical University.

### Plasmid constructs and RNA interference

The SNHG3 and STAT3 overexpressed plasmids were generated using the pCD513B lentiviral vector. The plasmids were transformed and then selected positive clones were cultured, followed by plasmid extraction and confirmation by sequencing. A medium containing 2 μg/mL puromycin was used to culture cells for 14 days to establish stable cell lines. In the case of siRNA transfection, cells were transfected with siRNAs using RNAiMAX (Invitrogen) and incubated for 48 hours. The siRNAs and inhibitors used in the experiments were purchased from GeneChem (China).

### Cell culture and selection

For this study, normal human breast epithelial cells (MCF-10A) and various BC cell lines (T47D, MDA-MB-231, MDA-MB-468, MCF7, BT-549, SK-BR-3) were provided by the American Type Culture Collection (USA). These BC cells were routinely cultured in a Forma™ direct heat CO_2_ incubator (Thermo Fisher Scientific, USA) under conditions of 37° C and 5% CO_2_. MDA-MB-468 and MDA-MB-231 cells were maintained in the L-15 medium, whereas SK-BR-3 cells were cultured in McCoy’s 5a medium. DMEM was used for culturing MCF7 and T47D cells, and RPMI1640 medium was employed for BT-549 and MCF-10A cells. The culture media were supplemented with 10% fetal bovine serum and 1% penicillin-streptomycin solution (100 U/ml penicillin, 0.1 mg/ml streptomycin). The flasks containing culture cells were placed in a 37° C incubator with 5% CO2, and the lids were loosely capped. When the confluence level of the cells reached over 80%, they were either passaged or frozen as required. The expression of SNHG3 in these cell lines was measured using qRT-PCR, and two cell lines with high SNHG3 expression were chosen for further experiments.

### Cell proliferation assay

Cell proliferation toxicity assays were performed using CCK-8 (Beyotime, China). Approximately 500 cells per well were seeded in 96-well plates for the control and experimental groups. Following overnight incubation of the cells mentioned above, CCK-8 reagent (10 μl per well) was added simultaneously to the culture wells for five consecutive days and incubated for 4 hours. Then, the absorbance was recorded at a wavelength of 450 nm on a 96-well plate enzyme marker, and the absorbance values were proportional to the number of cells proliferating in the culture. To conduct the colony formation assay, the cells mentioned above (at a concentration of 2.5×10^3) were seeded into a six-well plate and cultured for two weeks following the treatment. Subsequently, the cells were fixed with 100% methanol for 15 minutes, washed, and stained with a 0.1% crystal violet staining solution (Beyotime, China).

### Cell invasion and migration assay

Transwell chambers were positioned in a 24-well plate, and each compartment was coated with 50 μg of Matrigel gel. A cell suspension without serum, with a concentration of 5×10^5 cells/ml, was introduced into each chamber, amounting to 200 μl. McCoy’s 5A medium and 10% FBS were introduced into the 24-well plate (migration assays do not require spreading substrate gel). Following a 24-hour incubation period, the unperforated cells present on the surface of the chambers were gently removed using cotton swabs. Afterwards, the residual cells were fixed using methanol and subjected to staining with 0.1% crystal violet. Cells on the surface of the lower chamber of the filter membrane were then counted under an Olympus FSX100 microscope (Olympus, Japan) from a random count of 5 fields of view for each small chamber. The average number of stained cells was taken for each area of view.

### Cell cycle detection

Cell cycle assays were conducted using a Cell Cycle Kit (KeyGEN) following the provided instructions. In cell cycle experiments, we evaluated DNA binding to the fluorescent dye PI in the nucleus. The varying amounts of cellular DNA in each phase resulted in different levels of PI binding, which corresponded to different phases (G0/G1, S, and G2/M phases) based on the fluorescence intensity of the cells. Cell cycle analysis was performed using a FACSCalibur flow cytometer (BD Biosciences, USA).

### Establishment of a transplantation nude mice tumor model

MDA-MB-231 cells stably transfected with the sh-SNHG3 plasmid were cultured under standard conditions until they reached the exponential growth phase. After enzymatic digestion, the cells were detached, and the cell concentration was adjusted to 2.5×10^7 cells/ml. Following this, 5×10^6 cells were injected into the bilateral axillary regions of BALB/c nude mice in each experimental group. The long diameter (a) and short diameter (b) of the tumors in the mice were measured weekly. The tumor volume was calculated using the formula V=ab^2/2. This allowed us to generate a growth curve representing the changes in tumor size over time. We sacrificed the nude mice, weighed the transplanted tumor mass, and calculated the tumor suppression rate after six weeks. Mice were purchased from Yangzhou Medical College.

### Construction of nude mice tail vein tumor cell metastasis model

BALB/c nude mice aged 4-6 weeks were injected with suspensions of MDA-MB-231 cells stably transfected at a 2×10^6 cells/ml cell concentration into the tail vein. Each injection volume was 0.2 ml. At the end of 8 weeks, the mice were euthanized, and their lungs were dissected to assess and quantify the size and quantity of metastatic nodes. Then, the metastases samples were fixed, pathological sections were prepared, and the model was judged by pathological H&E staining analysis of the tumor.

### RNA pull-down assay

Briefly, the procedure involved obtaining probe-coated beads by incubating the SNHG3 probe with C-1 magnetic beads (Life Technology, USA). These beads were treated overnight with sonicated BC cells at four ° C, followed by elution and subsequent qRT-PCR analysis.

### RIP assay

The Magna RIPTM RNA Binding Protein Immunoprecipitation Kit (Millipore, USA) and AGO2 antibody (10686–1-AP, Proteintech, China) were utilized to perform RIP assays according to the instructions of the manufacturer. BC cells were subjected to RIP using the AGO2 antibody, and subsequent analysis involved measuring the relative expression of SNHG3 using qRT-PCR. The Ct value of the RIP fraction was normalized to that of the input RNA fraction.

### Western blot assay

BC cells were lysed using RIPA buffer (KeyGEN, China) containing a protease inhibitor to extract total protein. The BCA method determined the protein concentration (KeyGEN, China). The films were developed using the ECL analysis method after conventional immunoblotting steps, such as electrophoresis, membrane transfer, blocking, and antibody incubation. This analysis aimed to assess the expression of proteins related to growth and metastasis (CDK4, CDK6, Cyclin D1, N-Cadherin, Vimentin, MMP9) in cells. GAPDH protein was used as an internal reference for optical density analysis.

### Chromatin immunoprecipitation assay

EZ-Magna ChIP kit (Millipore, USA) was used to conduct ChIP. In summary, BC cells were initially exposed to a 1% formaldehyde solution for 15 minutes for fixation, followed by a 5-minute incubation with 125 nM glycine. Next, DNA fragments in the 200 to 300 bp range were created using sonication and underwent enzymatic digestion. Immunoprecipitation was performed using antibodies such as anti-STAT3 (#9139, Cell Signaling Technology, USA) and IgG. Finally, the precipitated DNA was analyzed using qPCR.

### Gene set enrichment analysis

To investigate the pathways and gene sets linked to the knockdown of SNHG3 in BC, Gene Set Enrichment Analysis (GSEA) was performed. GSEA v3.0 was utilized to determine if the gene members from the MSigDB database showed random distribution among the top or bottom ranks [16].

### Statistical analysis

Statistical analyses were performed using GraphPad Prism 7 (GraphPad, USA) and SPSS 19.0 (SPSS, USA) software packages. The distribution of clinical variables was studied using the chi-square test, and the Student’s t-test was used to analyze differences in gene expression levels. Survival analysis was determined using the Kaplan-Meier method, and differences were assessed using the log-rank test. The t-test or analysis of variance (ANOVA) was applied to evaluate group differences in both *in vitro* and *in vivo* experiments. The results are expressed as mean ± standard deviation (SD) from at least three independent replicates. Two-sided P-values were calculated and written, with significance indicated as P < 0.05, ** representing P < 0.01, *** representing P < 0.001, and “ns” indicating no statistical significance.

### Data availability statement

The original contributions presented in the study are included in the article material; further inquiries can be directed to the corresponding author.

## Supplementary Material

Supplementary Figures
