# Supplementary material for: lncSNHG3 drives breast cancer progression by epigenetically increasing CSNK2A1 expression level
Source: Aging (Albany NY). 2023 Jun 21;15(12):5734–50. doi: 10.18632/aging.204824 (PMC10333090; doi:10.18632/aging.204824)
Supplement: Supplementary Figures [file aging-15-204824-s001.pdf]

SUPPLEMENTARY FIGURES

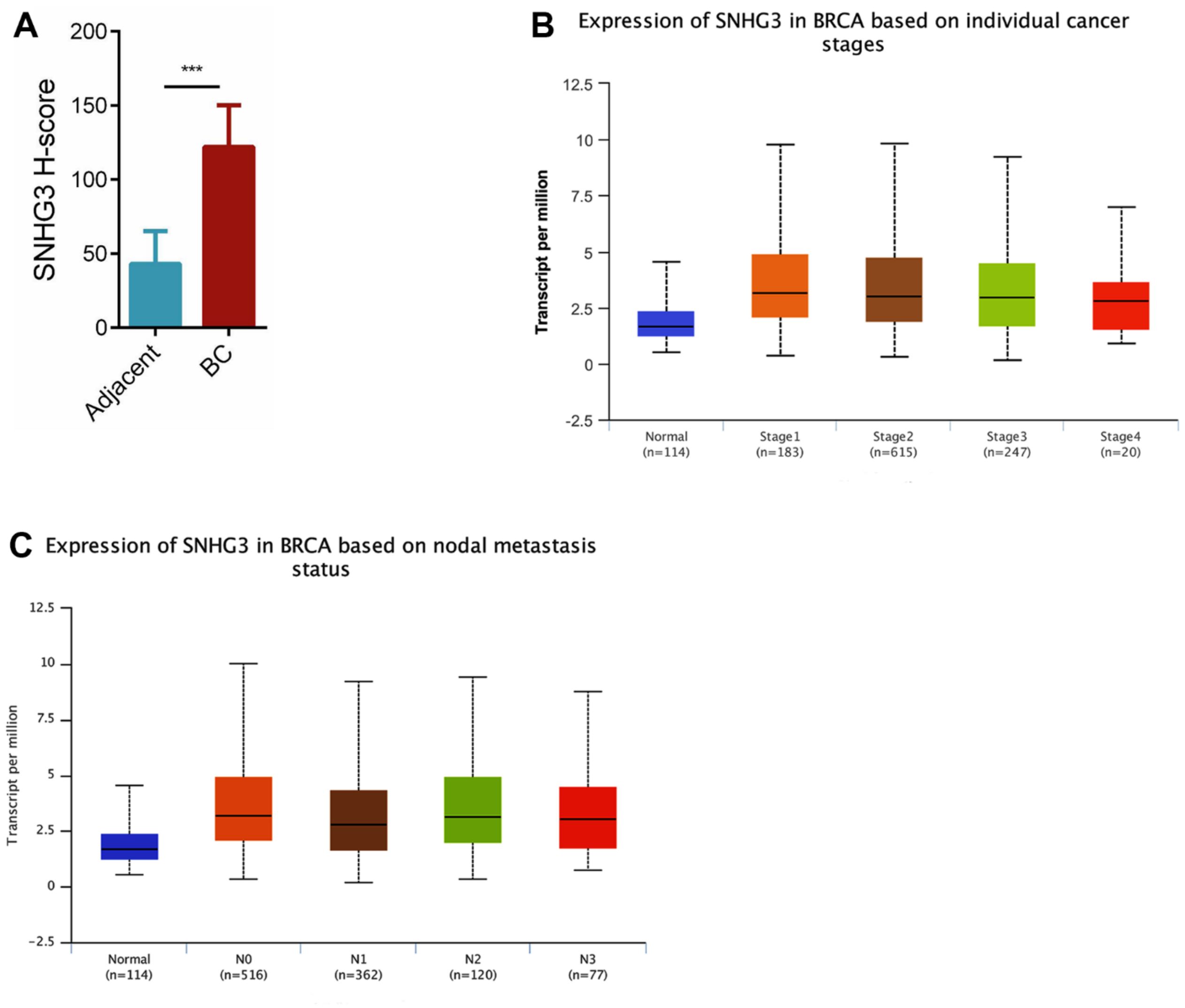

Supplementary Figure 1. (**A**) Statistical analysis results of SNHG3 expression level in BC tissues detected by ISH. (**B**) Expression levels of SNHG3 in BC tissues with different stages. (**C**) Expression levels of SNHG3 in BC tissues with different metastasis status.

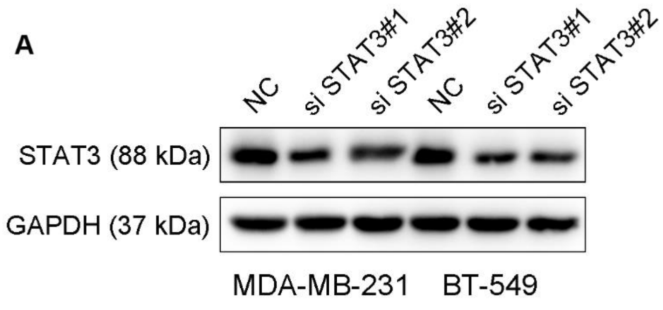

Supplementary Figure 2. (**A**) Knockdown efficiency of STAT3 was detected by Western blot.

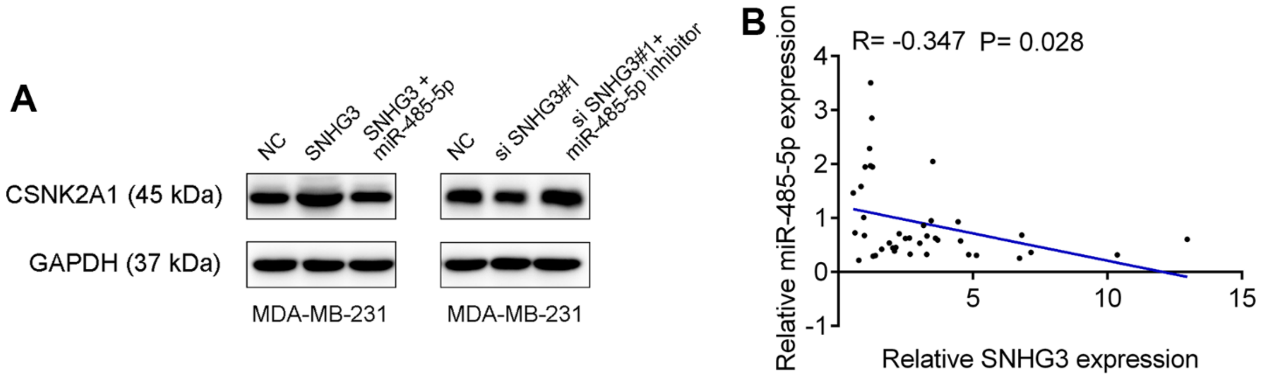

Supplementary Figure 3. (**A**) Protein level of CSNK2A1 with corresponding treatment conditions. (**B**) The correlation between the CSNK2A1 and miR-485-5p expression levels were analyzed in 40 paired BC samples.
